# Investigating the impact of key algorithm parameters and patient‐specific factors on the accuracy of CT ventilation imaging

**DOI:** 10.1002/acm2.70610

**Published:** 2026-05-14

**Authors:** Jeremy Lim, John Kipritidis, Jeremy T. Booth, Paul J. Keall, Hilary L. Byrne

**Affiliations:** ^1^ Image X Institute, Faculty of Medicine and Health The University of Sydney Sydney Australia; ^2^ Northern Sydney Cancer Centre Royal North Shore Hospital St Leonards Australia; ^3^ Institute of Medical Physics, School of Physics University of Sydney Sydney Australia; ^4^ Faculty of Medicine and Health The University of Sydney Sydney Australia

**Keywords:** algorithm parameters, CT ventilation imaging, sensitivity analysis

## Abstract

**Background:**

Computed Tomography Ventilation Imaging (CTVI) is an investigational technique that has its basis in functional lung avoidance radiotherapy. It offers a cost‐effective and accessible alternative to nuclear medicine imaging by generating lung ventilation maps from 4DCT or paired inhale/exhale breath‐hold CT (BHCT) scans. Despite over a decade of clinical validation, there is still no consensus on how algorithm parameters and patient‐specific factors influence CTVI accuracy. Further research is needed to understand CTVI's sensitivity to these variables and to standardize its implementation for clinical use.

**Purpose:**

This study evaluates how key algorithm parameters and patient‐specific factors affect the accuracy of CTVI.

**Materials and methods:**

CT ventilation images were generated from BHCT scans and compared to Galligas PET ventilation scans. The VESPIR toolkit was used to compute ventilation based on deformable image registration (DIR) evaluation of volume change (CTVI_Jac_) or change in Hounsfield Unit (HU) value (CTVI_HU_). CTVI accuracy was characterized as the voxel‐wise Spearman correlation (r_S_) with Galligas PET. Algorithm parameters common to many CTVI implementations were investigated with a baseline determined from existing literature: lung segmentation threshold (−600 HU to −150 HU), DIR regularization parameter (λ = 0.05 to 100), and smoothing filter diameter (0 voxels to 9 voxels). Robust parameter ranges were defined as those yielding r_S_ within 10% of the maximum cohort average observed through parameter variation, and no negative Jacobian values for the registration. Patient‐specific lung volume and density metrics were also analyzed to explain inter‐patient variability in CTVI accuracy.

**Results:**

The correlation between CTVI and Galligas PET was demonstrated to be robust within identified parameter ranges: lung segmentation threshold −600 HU to −150 HU for CTVI_Jac_ and CTVI_HU_, DIR regularization parameter (λ) 1.25 to 5 for CTVI_Jac_ and CTVI_HU_, and smoothing filter diameter 0 to 9 voxels for CTVI_Jac_ and 7 to 9 voxels for CTVI_HU_. No significant correlation was found between the accuracy of CTVI_Jac_ and any patient‐specific lung volume or density parameters. Significant correlations were found between the accuracy of CTVI_HU_ and the percentage change in lung volume during inspiration (*r* = 0.72, *p* < 0.01) and the lung volume in the exhale phase (*r* = −0.63, *p* < 0.01). The correlation between CTVI_Jac_ and CTVI_HU_ was found to be strongly correlated to CTVI accuracy.

**Conclusions:**

CTVI accuracy was relatively stable across the range of parameter values tested with no strong indication of the need for patient‐specific parameter sets. Patient‐specific differences appear to be a driving factor for inter‐patient variability in CTVI accuracy as parameter selection alone was insufficient to explain the variability. The strong association of CTVI_Jac_ and CTVI_HU_ agreement and CTVI accuracy suggests that CTVI_Jac_ and CTVI_HU_ agreement is a useful predictor of CTVI accuracy and quality metric for parameter optimization.

## 1 INTRODUCTION

Non‐contrast CT ventilation imaging (CTVI) is a cost‐effective and accessible method for visualizing lung ventilation compared to established techniques such as nuclear medicine and magnetic resonance imaging.

There is growing interest in using high‐ventilation regions identified by CTVI to guide radiotherapy planning. Vinogradskiy *et al*. demonstrated the feasibility of CTVI‐based functional avoidance radiotherapy in a multi‐institutional phase 2 trial, reporting a 10.1% reduction of symptomatic pneumonitis compared to a historical control.^1^ Baschnagel *et al.* similarly found reduced pulmonary toxicity and preservation of pulmonary function.^2^ Beyond radiotherapy, pathologically increased ventilation on CTVI has been found to be an early imaging marker for idiopathic pulmonary fibrosis.^3^, ^4^ The first commercial CTVI product, CT Lung Ventilation Analysis Software (4DMedical Limited, Melbourne), was approved in 2023. A systematic review of CTVI clinical validation studies found that further refinement and standardization of CTVI algorithms are required for implementation in prospective trials.^5^ A performance evaluation of the parameters affecting CTVI accuracy is therefore timely, particularly in the context of ongoing clinical trials investigating CTVI‐based functional avoidance treatment planning.

Most implementations of CTVI involve deformable image registration (DIR) of respiratory correlated images such as 4DCT or inspiration/expiration breath‐hold CT (BHCT) followed by estimation of ventilation from regional volume (Jacobian‐based) or density changes (Hounsfield Unit (HU)‐based).^6^

For clinical use, CTVI must be physiologically validated, ideally showing strong voxel‐level accuracy using Spearman's rank correlation coefficient (r_S_). Clinical human studies comparing CTVI against different reference modalities have shown variable mean r_S_ values of 0.37–0.40 (^99m^Tc‐DTPA SPECT),^7^ −0.02–0.26 (Technegas SPECT),^8^ 0.16–0.67 (Galligas PET),^9^, ^10^, ^11^ 0.37 (^3^He‐MRI),^12^ and 0.33 (^129^Xe‐MRI)^12^ which have been attributed to differences in image quality in the CT or comparator modality dataset, DIR implementation, and choice of ventilation metric.

Eslick *et al*.^9^ reported improvements in r_S_ of around 0.4 when CT ventilation images were derived from BHCT scans that were free of motion artifacts as opposed to 4DCT scans. Kipritidis *et al*.^10^ observed an improvement in r_S_ by 0.24 when using Galligas PET as the reference modality compared to earlier validation studies using ^99m^Tc‐DTPA SPECT which suffered from central airway depositions that yielded non‐representative ventilation. Even within related modalities, such as with hyperpolarized ^3^He and ^129^Xe MRI, r_S_ values can range from 0.4 to 0.8,^12^ reflecting inherent challenges in comparing ventilation imaging techniques that differ in underlying physiological processes or gas kinetics. There currently exists limited data on the level of accuracy needed for CTVI to be clinically useful. However, a study by Kida *et al*. gives an indication that an r_S_ of 0.4 and above between CTVI and nuclear medicine ventilation scans may be acceptable.^7^

CT ventilation images have been reported to be sensitive to differences in the degree of filtering applied to the CT ventilation images,^12^ DIR algorithm and ventilation metric,^13^ and image noise.^14^ The multi‐institutional VAMPIRE Challenge^15^ benchmarked multiple DIR algorithm and ventilation metric combinations using 4DCT scans with corresponding reference imaging (Galligas PET, DTPA SPECT or Xenon‐CT). A high degree of inter‐algorithm, inter‐subject and inter‐cohort variability in r_S_ was reported, with differences in breathing effort and scan quality contributing to inter‐subject variability. Several other studies have also investigated CTVI robustness with respect to acquisition‐related variables such as breathing amplitude in controlled experimental settings using mechanically ventilated animal models and repeat‐scan designs.^16^, ^17^

Understanding whether algorithm parameter selection or patient‐specific physiological factors contribute more substantially to CTVI variability is important for guiding commissioning effort and quality assurance in clinical trials.

This study aims to evaluate how key algorithm parameters and patient‐specific factors affect CTVI accuracy, measured by voxel‐wise Spearman correlation with Galligas PET.

## 2 METHODS

VESPIR (Ventilation, Segmentation and Pulmonary Image Registration), a MATLAB‐based toolkit developed by Kipritidis *et al*. was used for generating and validating the CT ventilation images.^18^ The toolkit was previously applied to 4DCT and BHCT images in validation studies comparing CTVI and nuclear medicine ventilation imaging.^9^, ^10^, ^15^, ^19^ A list of parameters within VESPIR that could potentially impact CTVI accuracy were identified, with parameters that were expected to have the most impact being selected for investigation in this study: lung segmentation threshold, DIR regularization parameter, and smoothing filter diameter.

A sensitivity analysis of CTVI accuracy to changes in the three key algorithm parameters was performed. Spearman correlation between the CT ventilation image and the corresponding Galligas PET was used as the primary metric to assess CTVI accuracy. This ranked correlation was chosen based on consistency with prior studies and for comparing images from two different modalities that are both interpreted in terms of ranking (e.g., high, medium and low ventilation relative to each patient's scan) rather than quantitative interpretation (e.g., using specific uptake values to determine high, medium and low ventilation). The percentage of negative Jacobian values and percentage decrease in mean square error (MSE) within the lungs are used as the primary metrics for registration quality and accuracy, adapted from the general recommendations of AAPM Task Group report TG 132.^20^

Robust parameter ranges were defined as a region of similar performance across the parameter sweep, identified using a heuristic criterion where mean r_S_ values were within 10% of the highest observed mean and no negative Jacobian values in the registration.

In this study, Galligas PET was treated as the ground truth for ventilation imaging. It offers several advantages over SPECT, including better spatial resolution, higher sensitivity, and smaller particle size (30–60 nm) which allows deeper penetration into airways. Drawbacks of Galligas PET include the logistical burden and cost of radionuclides, relatively low output of the ^68^Ge‐^68^Ga generator and short half‐life of ^68^Ga that makes it difficult to scan multiple patients in a single day.^21^

### 2.1 Image datasets

This study used a publicly available image dataset of 20 patients acquired at the Royal North Shore Hospital which was approved by the Human Research Ethics Committee (HREC/12/169) and registered in the Australian New Zealand Clinical Trials Registry (ACTRN12612000775819).^22^ Following the publication by Eslick *et al.*,^9^ the analyzed data consist of 16 successful inhale/exhale BHCT and corresponding Galligas PET scans. The BHCT scans for two patients failed due to their inability to comply with breath hold instructions (Patients 3 and 4). The scans were acquired using a Siemens Biograph mCT.S/64 PET/CT scanner (Siemens, Knoxville, USA) in one imaging session from lung cancer patients with ages between 54–73 years, lung cancer staging between II‐IV, and COPD grading from mild to severe. The voxel spacing of the BHCT and PET scans were 0.96 × 0.96 × 1.8 mm^3^ and 2.04 × 2.04 × 2.2 mm^3^, respectively.

High‐quality, radiologist‐verified lung masks of the exhale BHCT from a previous study, created using a semi‐automated algorithm with subsequent manual brushing to remove main airways, were also utilized in this work.^9^ Two patients (Patients 19 and 20) were excluded from this study as they did not include any radiologist‐verified lung masks.

### 2.2 CTVI generation and validation

Based on the multi‐institutional VAMPIRE challenge,^15^ the overall framework of VESPIR is representative of other published algorithms, although specifics such as the choice of DIR engine and type of image pre‐ and post‐processing might differ. The VESPIR workflow is presented here as a baseline case because it has been previously published and validated.^9^, ^10^, ^15^, ^19^

**FIGURE 1:**
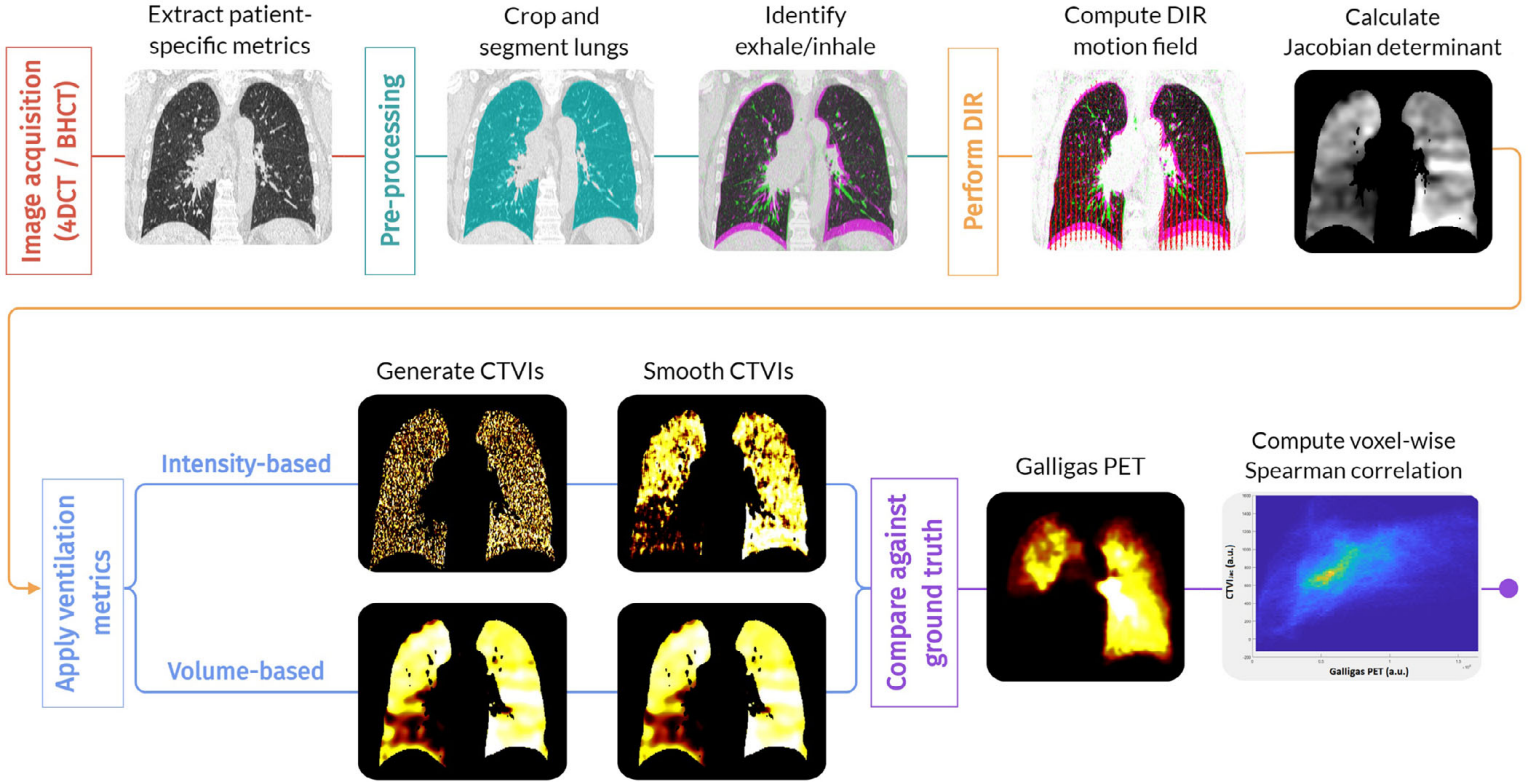
Flowchart showing the major stages in the CTVI pipeline: image pre‐processing, deformable image registration with calculation of the Jacobian determinant for DIR quality assurance, application of ventilation metrics, and comparison with an assumed ground truth.

A typical workflow within VESPIR for generating and validating CT ventilation images against the Galligas PET ground truth is illustrated in Figure 1.

In the VESPIR workflow, lung masks for the BHCT image pairs are created through an automatic segmentation process that first applies a median filter with a diameter of 3 voxels to remove noise, then binarizes the image according to a set lung intensity threshold (e.g., −500 HU).

A six‐stage DIR using Plastimatch v1.9.0 (http://plastimatch.org)^23^ B‐Spline (native implementation) is then performed to register the inhale BHCT to the exhale BHCT. The Plastimatch B‐Spline algorithm has been previously evaluated for the registration of thoracic CT, with a reported mean target registration error of 1.4 ± 0.2 mm in the VAMPIRE challenge and 1.40 mm in Phase 1 of the EMPIRE10 challenge.^15^, ^24^

Plastimatch performs iterative optimization of the deformation vector field (DVF) v with a cost function C(v) of the form:

$$C\;\left(v\right)=C_{\mathrm{sim}}\;\left(v\right)+\lambda C_{\mathrm{reg}}\left(v\right)$$

where $C_{\mathrm{sim}}$ is the similarity metric that penalizes images that do not match well, $C_{\mathrm{reg}}$ is the regularization term that penalizes unrealistic motion (e.g., not smooth, large amplitude) and λ, the DIR regularization parameter, is a scalar that controls the trade‐off between image similarity and smoothness of the DVF.^23^

A value for the DIR regularization parameter is set (e.g., λ=1) and a union mask of the inhale and exhale masks is created to limit the DIR cost function to voxels within the lung. Registrations that resulted in negative Jacobian values in the lungs, which imply a deformation that is not biologically plausible (tissue inversion), were excluded in accordance with the recommendation in the AAPM Task Group 132 report.^20^

The two main methods for calculating CT ventilation images are the Jacobian‐based method and the HU‐based method. The impact of parameter variation on both methods was investigated in this study.

The Jacobian‐based method (CTVI_Jac_) calculates the change in volume of each lung voxel from the Jacobian determinant of the DVF that maps between the inhale and exhale respiratory phases while the HU‐based method (CTVI_HU_) calculates the local change in air content for each lung voxel from the difference in HU between the deformed inhale and exhale phases.^25^, ^26^

For CTVI_Jac_, the following equation introduced by Reinhardt *et al*.^26^ was used:

$$\mathrm{CTV}I_{\mathrm{Jac}}\left(x\right)=\;\mathrm{Jac}\left(x\right)-1$$

where Jac(x) is the Jacobian determinant of the exhale‐to‐inhale DIR motion field,

$$\mathrm{Jac}\left(x\right)=\left|\begin{array}{ccc} 1+\frac{\partial u_{x}\left(x\right)}{\partial x} & \frac{\partial u_{x}\left(x\right)}{\partial y} & \frac{\partial u_{x}\left(x\right)}{\partial z}\\ \frac{\partial u_{y}\left(x\right)}{\partial x} & 1+\frac{\partial u_{y}\left(x\right)}{\partial y} & \frac{\partial u_{y}\left(x\right)}{\partial z}\\ \frac{\partial u_{z}\left(x\right)}{\partial x} & \frac{\partial u_{z}\left(x\right)}{\partial y} & 1+\frac{\partial u_{z}\left(x\right)}{\partial z} \end{array}\right|\;.$$

For the CTVI_HU_, a modified version of the equation by Guerrero *et al*.^25^ incorporating voxel‐wise tissue density scaling was used:

$$\mathrm{CTV}I_{\mathrm{HU}}\;\left(x\right)=\frac{HU_{\mathrm{ex}}\left(x\right)-{\mathrm{HU}}_{\mathrm{in}}^{*}\left(x\right)}{{\mathrm{HU}}_{\mathrm{in}}^{*}\left(x\right)+1000}\;\rho_{\mathrm{scaling}}\left(x\right)$$

where $HU_{\mathrm{ex}}\left(x\right)$ and ${\mathrm{HU}}_{\mathrm{in}}^{*}\left(x\right)$ are the CT numbers at voxel x in the exhale image and the deformed inhale image, respectively, while the asterisk represents a global intensity correction that is applied to the deformed inhale image to account for changes in lung mass due to changes in blood distribution during inspiration.^11^ $\rho_{\mathrm{scaling}}\;\left(x\right)=\left(HU_{\mathrm{ex}}\left(x\right)+1000\right)\;\;/\;1000$ was applied to improve modeling of radioaerosol deposition when correlating CTVI against nuclear medicine ventilation imaging.^10^

The generated CT ventilation images were defined on the exhale BHCT and smoothed with an edge‐preserving median filter. The choice of smoothing filter diameter, such as the 3‐voxel diameter median filter employed in the VAMPIRE challenge, impacts the trade‐off between noise reduction and spatial fidelity.^15^

Validation was performed by resampling the CT ventilation images to match the spatial dimensions of the Galligas PET images using nearest‐neighbor interpolation. Voxel‐wise Spearman correlations between the CT ventilation images and Galligas PET image were calculated on lung voxels that are present in both the high‐quality, radiologist‐verified lung mask and coarse lung mask generated by VESPIR.

A baseline set of parameter values was chosen based on previously published implementations of VESPIR from Eslick *et al*. and the VAMPIRE challenge (Table 1).^9^, ^15^ The parameter investigation was scripted using VESPIR modules, with a single parameter being varied from the baseline each time.

**TABLE 1:** Baseline parameter values for generating CT ventilation images.

Parameter	Value
Lung segmentation threshold	−250 HU
DIR regularization parameter	1
Smoothing filter diameter	3 voxels (2.88 × 2.88 × 5.4 mm^3^)

### 2.3 Variation of lung segmentation threshold

Thresholds of −200 HU,^27^ −250 HU,^25^, ^28^ −400 HU^29^ and −500 HU^18^ have been used in literature to segment lung parenchyma. The choice of lung segmentation threshold impacts the extent of inclusion and exclusion of specific features such as vessels and ground‐glass opacities which in turn impacts the performance of the DIR and voxels that are included in the Spearman correlation calculation. Extending beyond the range of values from literature cited above, 10 lung masks, corresponding to segmentation thresholds between −600 HU and −150 HU with a 50 HU interval, were generated and used to perform DIR for each patient.

### 2.4 Variation of DIR regularization parameter λ

**FIGURE 2:**
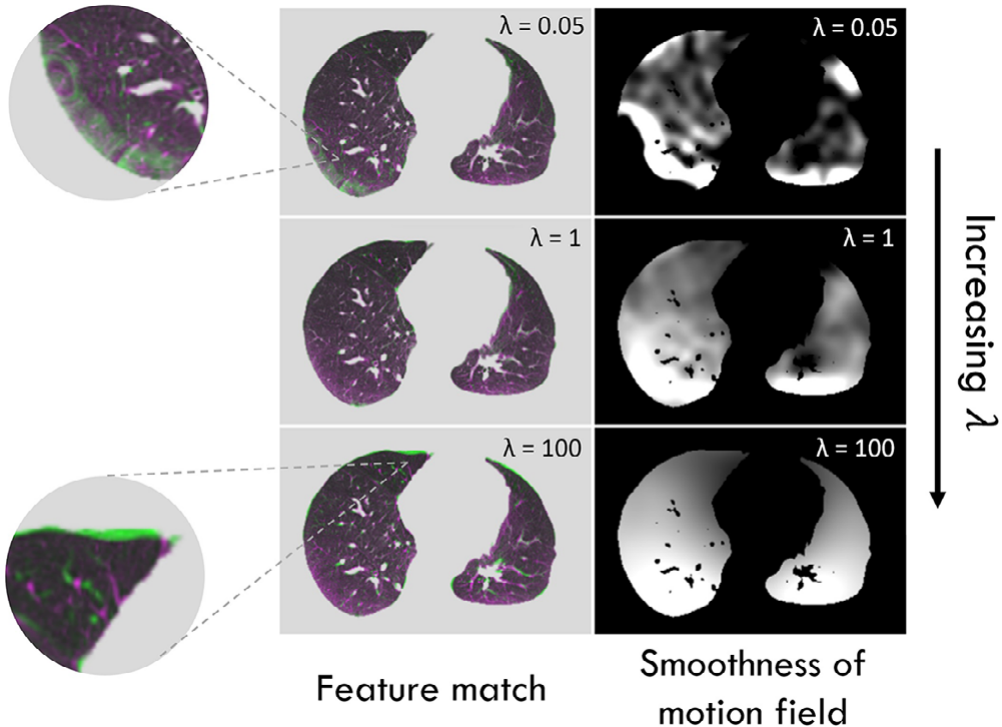
Feature match between deformed inhale BHCT and exhale BHCT (left column) and Jacobian determinant of the DVF (right column) at λ values of 0.05, 1, and 100 (top to bottom) for a representative case.

18 different values of DIR regularization parameter λ ranging logarithmically from 0.05 to 100 were used to perform registrations for each patient. The range of values was selected to cover the two extremes of feature matching and smoothness of the motion field (Figure 2).

The registration quality and CTVI accuracy were assessed using four metrics: percentage of negative Jacobian values in the lung, MSE between the deformed inhale and exhale image, Spearman correlations r_S_ between CTVI_Jac_ and Galligas PET, and also between CTVI_HU_ and Galligas PET.

### 2.5 Variation of smoothing filter diameter

Smoothing of CT ventilation images helps to mitigate some of the inaccuracies in registration but it is important to strike a balance between noise reduction and preserving spatial accuracy. In this experiment, ventilation images were produced for each patient using baseline parameters for lung segmentation and DIR. The CT ventilation images were then smoothed using four different median filter diameters ranging from 0 to 9 voxels, and the resulting images were assessed by Spearman correlation to Galligas PET. Median filtering was chosen to preserve edges and prevent undesirable smearing between voxels in lung and non‐lung regions.

### 2.6 Patient‐specific lung volume and density parameters

Various indices were extracted from the BHCT scans to investigate their correlation with CTVI accuracy. Whole‐lung volume and mean HU, percentage of lung regions indicative of air trapping (< −850 HU),^30^ absolute and percentage change in lung volume between the inhale and exhale BHCTs, and coefficient of variation, which characterizes image heterogeneity, were calculated. To quantify the strength of the linear relationship, the Pearson correlation coefficient was used.

### 2.7 Evaluating a CTVI quality metric for parameter optimization

For CTVI to become more widespread and implemented in the clinic, there is a need for a metric to assess the quality of the ventilation images in the absence of a ground truth. The correlation between CT ventilation images and clinical modalities has been observed to be higher when the paired CTVI_Jac_ and CTVI_HU_ correlated more strongly with each other.^15^ In this investigation, we evaluate the use of this self‐correlation for parameter optimization by examining how well it tracks with CTVI accuracy in response to changes in the DIR regularization parameter.

## 3 RESULTS

### 3.1 Impact of lung segmentation threshold

**FIGURE 3:**
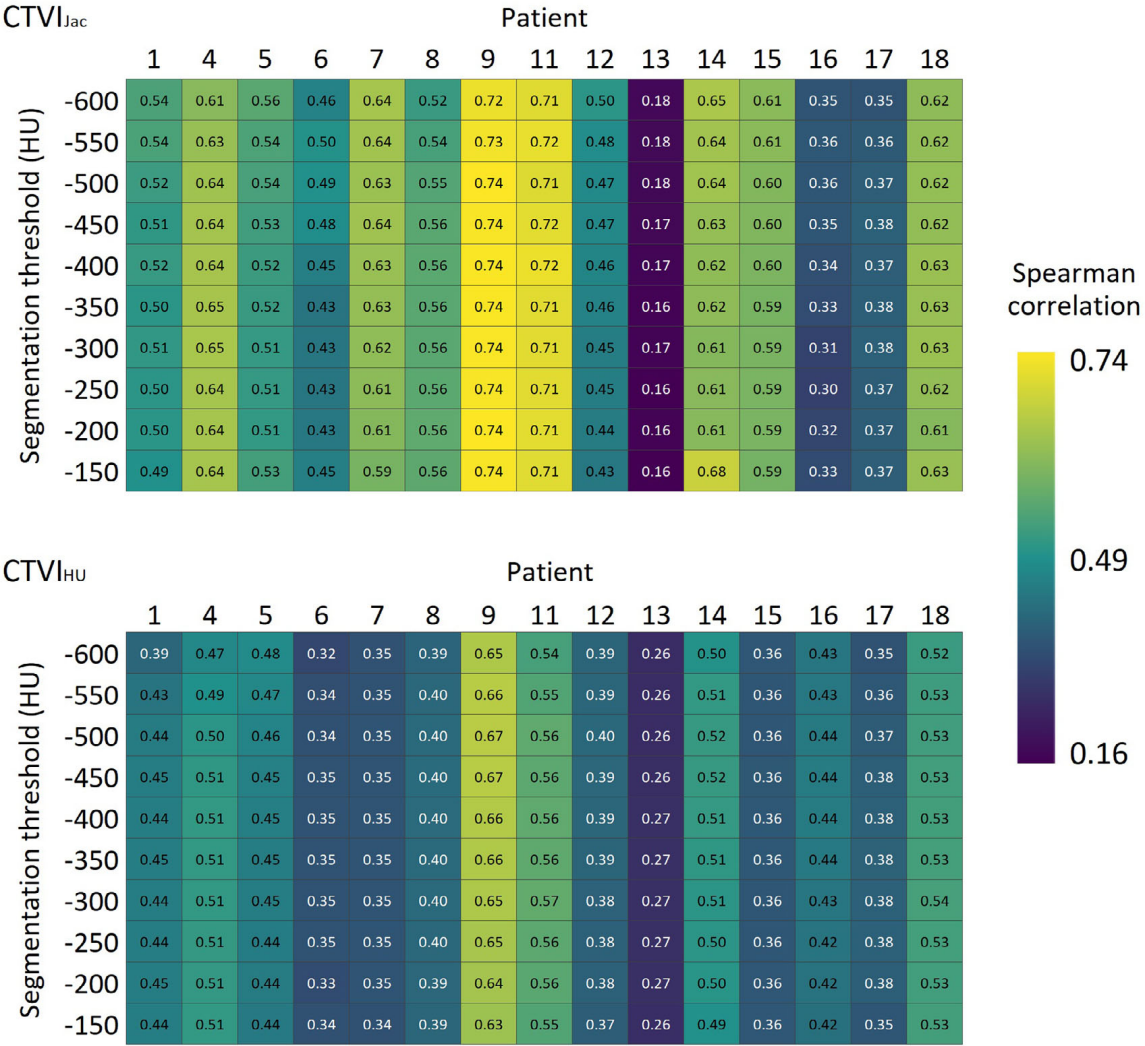
Heat map of the Spearman correlations between CTVI_Jac_ (top) or CTVI_HU_ (bottom) and the corresponding Galligas PET scans for different lung segmentation thresholds.

Figure 3 shows a heat map of the Spearman correlations for each patient and segmentation threshold investigated. As the lung segmentation threshold was varied between −600 HU and −150 HU, the mean r_S_ ranged from 0.52 to 0.54 for CTVI_Jac_ and 0.43 to 0.44 for the CTVI_HU_. Figure 3 reveals that the variation in r_S_ was predominantly patient‐specific, with the per‐patient mean r_S_ across the thresholds ranging from 0.17 to 0.74 for CTVI_Jac_ and 0.26 to 0.65 for CTVI_HU_.

The lung segmentation thresholds that resulted in the highest mean r_S_ forCTVI_Jac_ and CTVI_HU_ were −550 HU and −450 HU, respectively. Paired *t*‐tests found no significant difference (*p* > 0.05) in the mean r_S_ between the best performing threshold and thresholds in the range of −400 HU to −500 HU for both CTVI_Jac_ and CTVI_HU_.

### 3.2 Impact of DIR regularization parameter λ

For values of λ ≥ 1, zero negative Jacobian values were observed within the lungs for all patients. Negative Jacobian values started to appear when λ < 1 and the percentage of negative Jacobian values increased at lower values of λ. The MSE between the deformed inhale and exhale images increased monotonically with λ, which was expected as λ directly controls the trade‐off between image similarity and spatial smoothness of the motion field.

As λ was varied from 0.05 to 100, the mean r_S_ for CTVI_Jac_ and CTVI_HU_ ranged from 0.25–0.60 and 0.36–0.45, respectively, and displayed a similar trend of rise and fall, reaching a maximum at λ = 10 and λ = 0.5, respectively.

**FIGURE 4:**
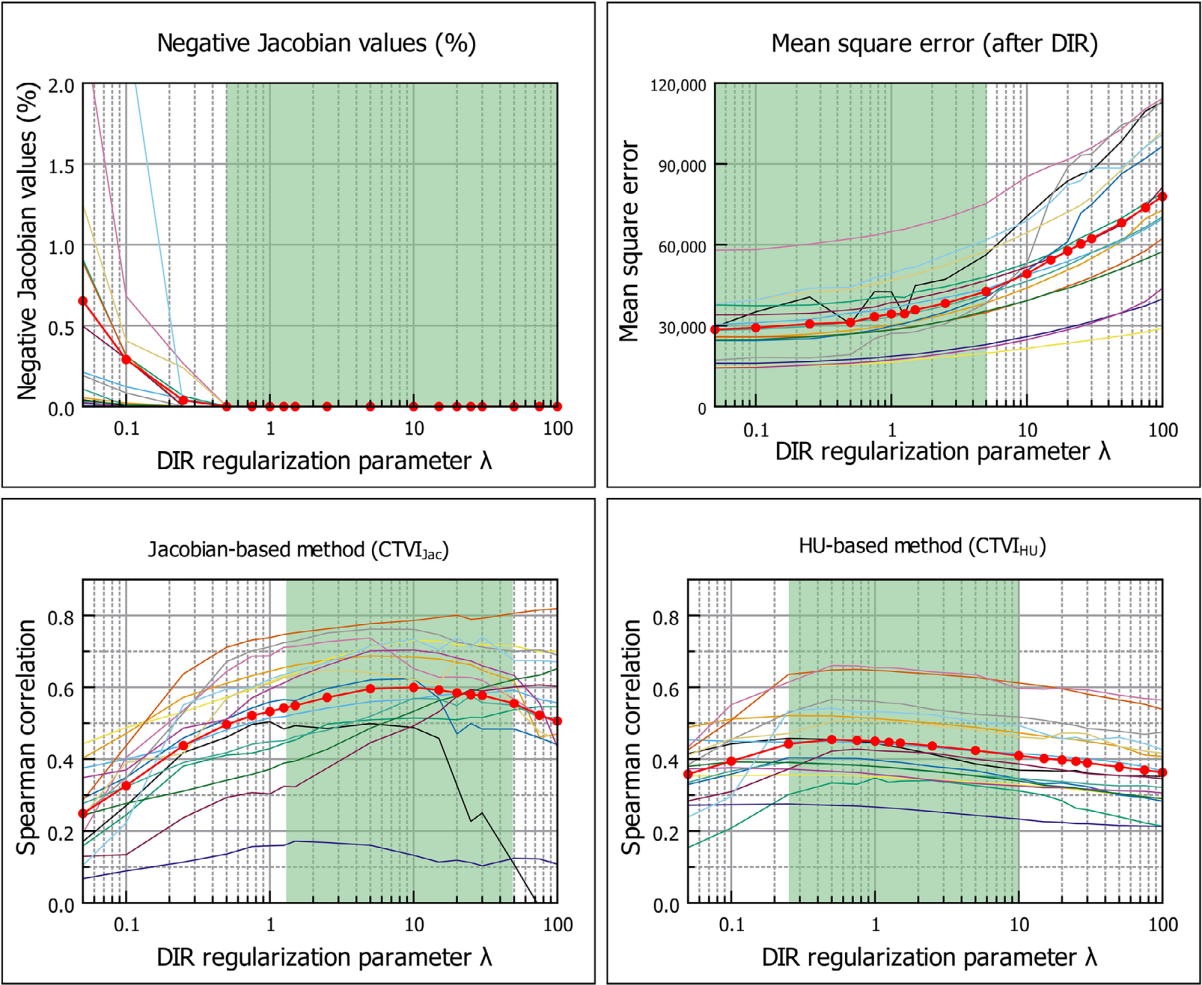
Variation of DIR and CTVI accuracy metrics with DIR regularization parameter. Individual patient results are shown as thin colored curves, with the cohort mean shown as a thick red curve. Green bands indicate regions of good performance. The green bands of the four metrics overlap where 1.25 ≤ λ ≤ 5.

λ values between 1.25 and 5 were found to give robust results, as shown in Figure 4. The range was determined by the absence of negative Jacobian values, visual assessment of regions where MSE was relatively stable, and mean r_S_ that were within 10% of the maximum for each CTVI method.

### 3.3 Impact of smoothing filter diameter

**FIGURE 5:**
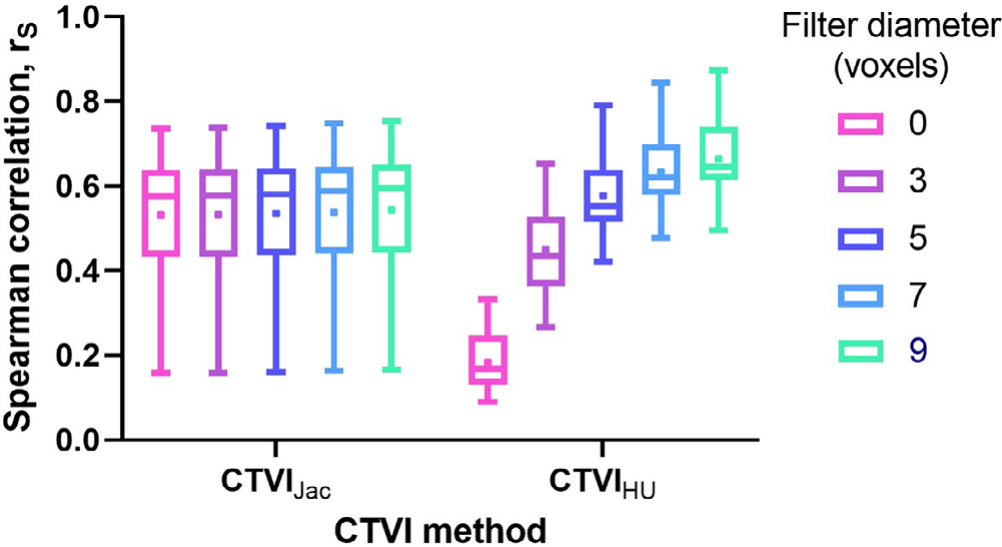
Box plots of the Spearman correlations (r_S_) between each type of CTVI and corresponding Galligas PET scans, with mean r_S_ shown as dots, for different median filter diameters (3 voxels = 2.88 × 2.88 × 5.4 mm^3^, 5 voxels = 4.80 × 4.80 × 9.0 mm^3^, 7 voxels = 6.72 × 6.72 × 12.6 mm^3^, 9 voxels = 8.64 × 8.64 × 16.2 mm^3^).

For CTVI_Jac_, there was negligible improvement in r_S_ with increasing filter diameter, with mean r_S_ ranging from 0.53 to 0.54. For CTVI_HU_, the mean r_S_ significantly improves with larger filter diameters, with mean r_S_ ranging from 0.18 to 0.67. CTVI_HU_ also outperforms CTVI_Jac_ in terms of mean r_S_ at filter diameters ≥5 voxels, as shown in Figure 5. Paired *t*‐tests found that median filtering the CT ventilation images always led to statistically significant improvement in r_S_ in both methods (*p* < 10^−4^).

### 3.4 Impact of patient‐specific lung volume and density parameters

For CTVI_Jac_, no significant correlation (*p* < 0.05) was found between any patient‐specific factor and CTVI accuracy. For CTVI_HU_, percentage change in lung volume, lung volume at peak exhale, and absolute change in lung volume were significantly correlated with CTVI accuracy. The corresponding Pearson correlation coefficients, 95% confidence intervals, and *p*‐values are summarized in Table 2.

**TABLE 2:** Pearson correlations (r_P_) between various patient‐specific factors and CTVI accuracy_._

	CTVI_Jac_		CTVI_HU_	
Patient‐specific factor	r_P_ (95% CI)	*p*‐value	r_P_ (95% CI)	*p*‐value
Percentage change in lung volume	0.36 (−0.17–0.73)	0.17	0.72 (0.34–0.90)	< 0.01
Lung volume at peak exhale	−0.16 (−0.61–0.37)	0.56	−0.63 (−0.86–0.20)	< 0.01
Absolute change in lung volume	0.36 (−0.17–0.72)	0.18	0.52 (0.04–0.81)	0.04
Percentage of air trapping (< −850 HU)	−0.12 (−0.58–0.40)	0.67	−0.50 (−0.80–0.00)	0.05
Mean HU	0.12 (−0.40–0.58)	0.66	0.47 (−0.03–0.78)	0.07
Coefficient of variation	−0.36 (−0.73–0.16)	0.17	−0.40 (−0.75–0.12)	0.12
Lung volume at peak inhale	0.04 (−0.46–0.53)	0.87	−0.34 (−0.71–0.19)	0.20

### 3.5 Parameter optimization using a CTVI quality metric

**FIGURE 6:**
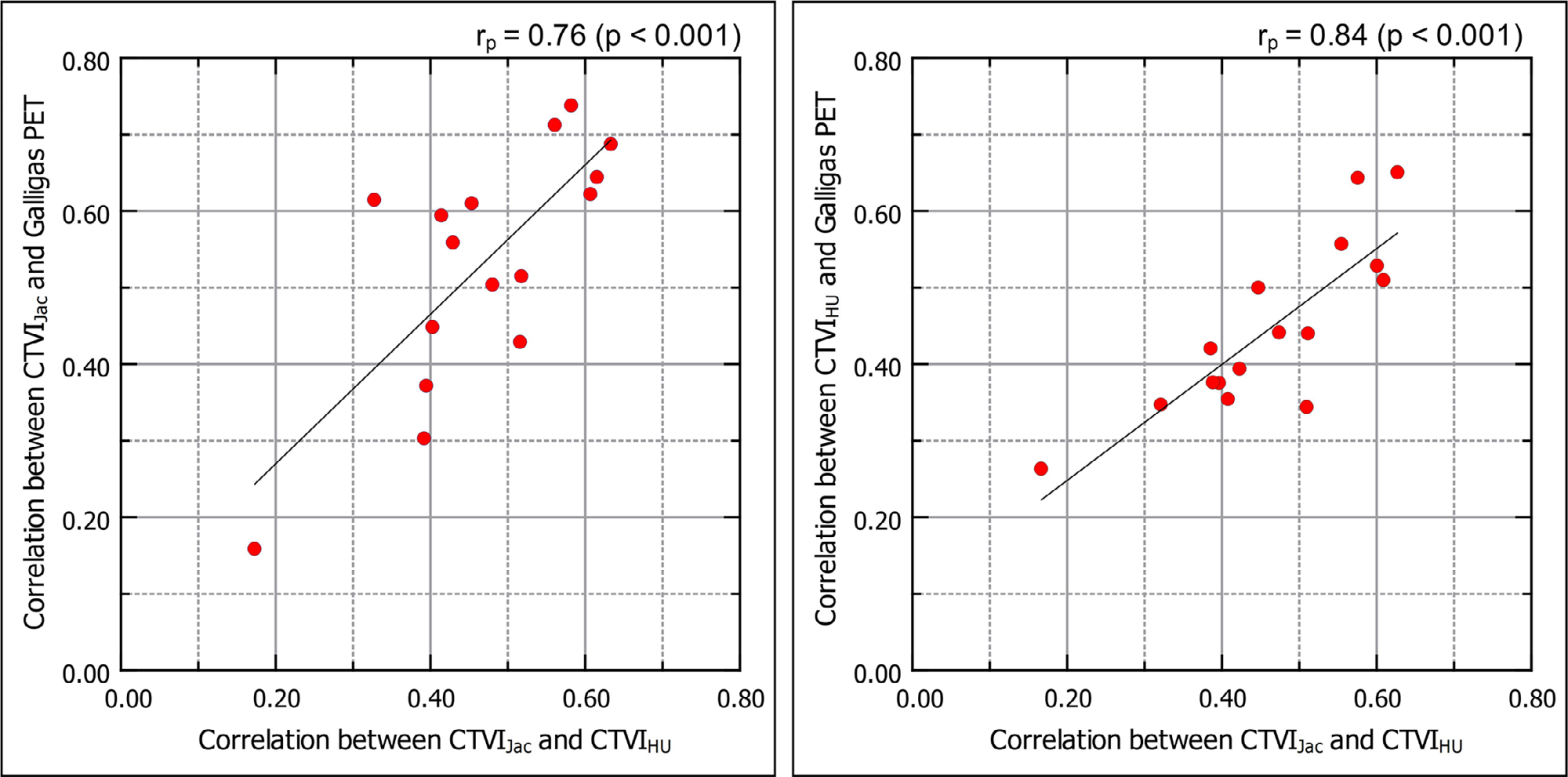
Relationship between CTVI accuracy and the correlation between paired CTVI_Jac_ and CTVI_HU_. The vertical axes show the Spearman correlation between CTVI_Jac_ (left) or CTVI_HU_ (right) and Galligas PET. The horizontal axes show the Spearman correlation between paired CTVI_Jac_ and CTVI_HU_. Pearson correlation coefficients (r_p_) between the variables are shown on the top right of each graph.

In this study, when the baseline set of parameters was used, strong Pearson correlations were observed between the correlation of paired CTVI_Jac_ and CTVI_HU_ and their respective accuracy against Galligas PET: r_P_ = 0.76 (*p* < 0.001) for CTVI_Jac_ and r_p_ = 0.84 (*p* < 0.001) for CTVI_HU_, as shown in Figure 6.

**FIGURE 7:**
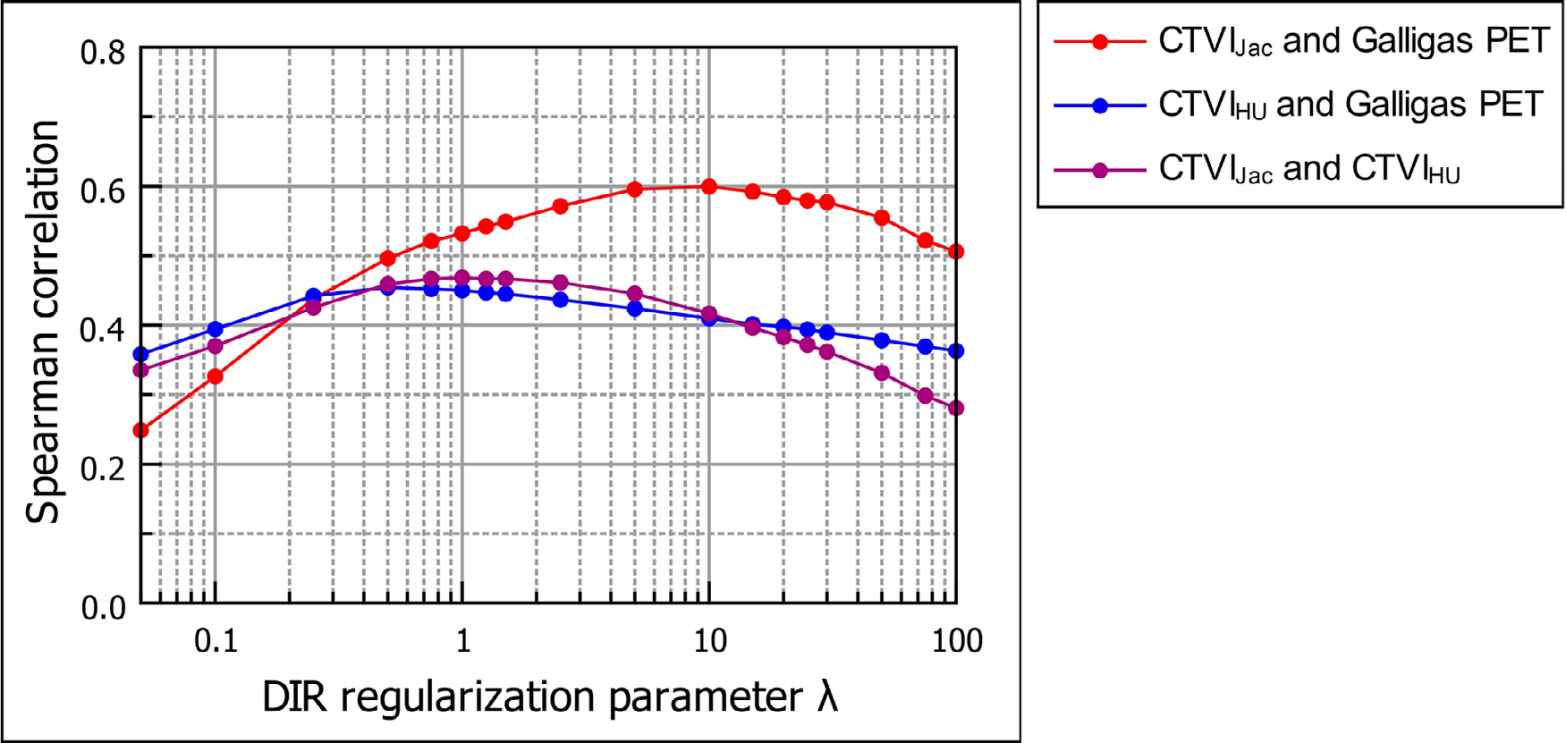
Spearman correlation of each corresponding pair of CTVI_Jac_ and CTVI_HU_ with Galligas PET and each other at different values of DIR regularization parameter λ.

The correlation between paired CTVI_Jac_ and CTVI_HU_ was observed to track well with CTVI accuracy in response to parameter variation, exhibiting a similar trend of rise and fall as the DIR regularization parameter λ was varied, as seen in Figure 7.

The mean correlation of paired CTVI_Jac_ and CTVI_HU_ reached a maximum at λ = 1. At this value of λ, the mean correlations of CTVI_Jac_ and CTVI_HU_ with Galligas PET, were 0.53 and 0.45, respectively. For comparison, the maximum mean correlations of CTVI_Jac_ and CTVI_HU_ with Galligas PET observed from variation of the DIR regularization parameter were 0.60 and 0.45, respectively.

## 4 DISCUSSION

An investigation of the sensitivity of CTVI accuracy to key algorithm parameters in the production pathway of a specific implementation of CTVI (VESPIR) using BHCT and compared to Galligas PET was performed. The main finding was that the correlation between CTVI and Galligas PET was robust to changes in individual parameters within identified parameter ranges: lung segmentation threshold from −600 HU to −150 HU for CTVI_Jac_ and CTVI_HU_, DIR regularization parameter λ from 1.25 to 5 for CTVI_Jac_ and CTVI_HU_, and smoothing filter diameter from 0 to 9 voxels for CTVI_Jac_ and 7 to 9 voxels for CTVI_HU_.

It is important to emphasize that all findings in this study are specific to the CTVI implementation, imaging protocol, and the studied cohort, and should not be assumed to generalize to other algorithms or imaging protocols without further validation. 4DCT or free‐breathing scans introduce phase‐sorting artifact and motion blurring, which can amplify sensitivity to the DIR regularization parameter. Scanner‐specific characteristics, including imaging dose and reconstruction algorithm may further influence the sensitivity to segmentation thresholds and smoothing.

The use of BHCT acquisition, DIR‐based ventilation estimation, and overall processing framework in this study are consistent with those employed in the VITaL trial (NCT06127654), supporting the practical relevance of this analysis in relation to ongoing functional lung avoidance studies. Within this context, the observed robustness suggests the potential to reduce the burden of clinical commissioning and patient‐specific quality assurance. That is, after identifying a set of parameters that is generally appropriate for a given combination of algorithm, modality, and cohort, then there may be less need or benefit in tuning those parameters separately for each patient, although this should be validated in larger and more diverse cohorts.

The impact of varying the lung segmentation threshold from −600 HU to −150 HU was small (< 0.02) changes in the mean r_S_ between CTVI and Galligas PET. A limitation of this investigation is that using a union mask for registration may have infilled some of the excluded parts of the lung, reducing the effect of varying the threshold.

The impact of varying the DIR regularization parameter λ from 0.05 to 100 was changes in the mean r_S_ by up to 0.35 and 0.10 for CTVI_Jac_ and CTVI_HU_, respectively. The greater sensitivity of CTVI_Jac_ to λ may reflect its direct dependence on the DIR motion field. This finding highlights the importance of identifying a stable range of λ as the observed level of variation could potentially have a clinical impact.

The impact of varying the smoothing filter diameter on CTVI_Jac_ was negligible, as Plastimatch produces an intrinsically smooth Jacobian image when the baseline value of λ = 1 is used, as seen in Figure 2. In contrast, CTVI_HU_ are more susceptible to image noise and registration inaccuracies due to the nature of voxel‐level difference calculations and hence benefit greatly from smoothing. It is important to emphasize that the need for smoothing does not imply that unsmoothed CTVI_HU_ are clinically irrelevant, but rather that it should be interpreted in the context of the spatial resolution and noise characteristics of CT and the reference modality. Galligas PET produces smoother, inherently lower‐resolution images that reflect particle deposition during tidal breathing and blurring from respiratory motion. Consequently, some degree of smoothing is necessary to match the spatial resolution of the two modalities. Without smoothing, voxel‐wise Spearman correlations would be dominated by small‐scale mismatches that are below the resolution of the Galligas PET ground truth.

A similar investigation by Tahir *et al.* evaluated the spatial correlation between CT ventilation images and hyperpolarized gas MRI (^3^He and ^129^Xe), reporting a marked improvement in CTVI_HU_ with increasing median filter radius. This improvement plateaued at a diameter of 6 voxels (5.88 × 5.88 × 15 mm), beyond which no statistically significant increase in correlations was observed.^12^ In contrast, statistically significant increases in Spearman correlations were observed at each increase in filter diameter in this study, with a much greater magnitude of improvement. These differences are likely due to differences in the spatial resolution of the reference modality and the physiological processes being captured, with hyperpolarized gas MRI offering finer detail and reflecting ventilation during a controlled breath‐hold.

In contrast to the finding that CTVI accuracy is robust to changes in algorithm parameters, there was large inter‐patient variability in the correlations, as seen in Figures 3 and 4. This variability may be due to patient‐specific differences in breathing maneuver and scan quality. In particular, a strong Pearson correlation of 0.72 (*p* < 0.002) was observed between the accuracy of CTVI_HU_ and the percentage change in lung volume between the inhale and exhale phases. One possibility for this observation is that with a larger lung volume change, the difference in HU between the inhale and exhale BHCTs is greater, resulting in a stronger signal‐to‐noise ratio. As lung volume change is a patient characteristic rather than a modifiable parameter, this relationship should not be interpreted as an optimization strategy but rather as a predictive quality factor that may indicate the expected reliability of CTVI. In practice, this suggests that a minimum lung volume change could serve as a practical threshold for reliable ventilation estimation.

One limitation of this study was that each investigation was carried out with only a single parameter being varied from a published baseline with an assumption of mutual exclusivity of parameters. While this sensitivity analysis is useful in providing a stable starting point within a specific implementation of CTVI, patient cohort and source image modality, these settings may not be generalizable to variations of the two main ventilation metrics, other cohorts, and comparisons to modalities other than Galligas PET. Nevertheless, several of the findings are generalizable. For instance, CTVI_HU_ is based on voxel‐level intensity differences that are subject to a high degree of noise and can benefit from filtering regardless of CTVI implementation. The robustness of CTVI to lung segmentation threshold is also applicable to other B‐spline‐based DIR algorithms. Moreover, the observed impact of the regularization parameter in this study likely extends to other DIR algorithms that incorporate a regularization term, as they are expected to exhibit a similar trade‐off between image similarity and spatial smoothness. The effect of increasing the strength of a regularization parameter such as λ in this study is to smooth out CTVI_Jac_, similar to how median filtering impacts CTVI_HU_. However, CTVI accuracy decreases when the motion field is overly constrained and the image becomes excessively smoothed. Sensitivity to parameters investigated in this study may vary substantially with DIR algorithms that are fundamentally different from B‐spline, such as diffeomorphic transforms or biomechanical methods. Nevertheless, the sensitivity analysis framework used here can guide the evaluation of parameter robustness in these alternative implementations.

Deep learning is increasingly used for the synthesis of CT ventilation images.^31^, ^32^, ^33^ While the performance of these models can be impacted by various image processing parameters, most existing literature has concentrated on mechanistic DIR‐based methods. As the present work aimed to compare like‐for‐like parameter sets, deep learning approaches were not included in this analysis.

The study is limited by a small, single‐institution cohort of 16 patients which restricts statistical power. Consequently, associations between CTVI accuracy and patient‐specific factors, such as lung volume changes, should be considered exploratory and would benefit from validation in larger, multi‐center studies. To our knowledge, the patient cohort dataset used in this study,^9^ available from The Cancer Imaging Archive, is the only open‐access dataset with both multi‐phase CT scans and independent ventilation images for the same patients. Therefore, this study is necessarily limited to this dataset. However, should new datasets become available, the sensitivity analysis method developed here could be applied to these new datasets.

The strong correlation observed between CTVI accuracy and the correlation between paired CTVI_Jac_ and CTVI_HU_ supports its investigation as a secondary check and quality metric for CTVI accuracy. When the correlation between paired CTVI_Jac_ and CTVI_HU_ was used to optimize the DIR regularization parameter, the resulting CTVI accuracy was 0.45 for CTVI_HU_, matching the maximum seen through parameter variation, and 0.53 for CTVI_Jac_, within 0.07 of its maximum of 0.60, demonstrating its suitability for CTVI parameter optimization in the absence of a ground truth.

## 5 CONCLUSION

For the implementation of CTVI used in this study, the Spearman correlation between CTVI and Galligas PET was found to be robust to changes in lung segmentation threshold and DIR regularization parameter with no strong indication of the need for patient‐specific parameter sets. The correlation between paired CTVI_Jac_ and CTVI_HU_ was found to be a useful predictor of CTVI accuracy and quality metric for parameter optimization.

Patient‐specific differences appear to be a driving factor for inter‐patient variability in CTVI accuracy as parameter selection alone was insufficient to explain the variability seen in this work.

## AUTHOR CONTRIBUTIONS

Jeremy Lim processed the images, analyzed the results, and wrote the manuscript. Paul Keall and Hilary Byrne conceived the original idea. Hilary Byrne and John Kipritidis further developed and planned the work that led to the study. All authors discussed the analysis methods and results, reviewed and revised the manuscript, and approved the final version.

## CONFLICT OF INTEREST STATEMENT

Paul Keall is an inventor of a patent on CT ventilation (#7668357). This patent and associated intellectual property have been licensed by the University of Sydney to 4DMedical Limited. John Kipritidis is a co‐inventor of a CT ventilation software that has been licensed by the University of Sydney to 4DMedical Limited.

## Data Availability

The dataset used in this study is available on The Cancer Imaging Archive under the title ‘CT vs PET Ventilation Imaging’.^22^
